# The attitudes of obstetric caregivers towards labour pain relief methods and associated factors at public health centers of East Gojjam zone, Amhara Region, Ethiopia: Institutional based cross-sectional study

**DOI:** 10.11604/pamj.2022.42.47.31439

**Published:** 2022-05-17

**Authors:** Keralem Anteneh Bishaw, Misganaw Fikrie Melesse, Bewket Yeserah Aynalem

**Affiliations:** 1 Department of Midwifery, Debre Markos University, Debre Markos, Ethiopia

**Keywords:** Attitude, labor pain relief, obstetric caregiver, Ethiopia

## Abstract

**Introduction:**

labour pain relief is a key factor for maternal satisfaction during childbirth. However, in developing countries, labour pain management is not a well-established service mainly due to negative attitudes of health care providers resulting in unmeasured suffering from childbirth for mothers. Thus, this study was aimed to assess attitude of obstetric caregiver towards labour pain management and associated factors at public health centers of East Gojjam zone.

**Methods:**

institutional-based cross-sectional study was conducted from March 1-30, 2018. Three hundred and nine sampled obstetric caregivers have participated, with a 96.8%(299) response rate. Data were collected with structured pretested questionnaires. Data were entered into Epi data 4.2 versions and bivariate and multivariate logistic regression was carried out using SPSS 23 versions with 95 % CI to determine the association between dependent and independent variables.

**Results:**

out of the study participants, 128 (42.8%) had a negative attitude towards managing labour pain. Knowledge (AOR =3.785, 95 % CI: 2.251,6.365), training (AOR=2.923, 95% CI: 1.266, 6.749) and Companion (AOR=1.834, 95% CI: 1.055, 3.189) had significantly associated with attitude of obstetric caregiver towards labour pain relief methods.

**Conclusion:**

the result of this study showed that there is still a negative attitude towards labour pain management among obstetric caregivers in the study setting. Providing knowledge-based in-service training for obstetric caregivers to change their attitude towards labour pain relief methods is advisable.

## Introduction

Labour pain is physiological pain that occurs during labour progress and natural processes taking place in the women´s body [1]. It is the most severe pain for females and inevitable aspects of the childbirth process, but different from other pain. It is not a sign of injury or tissue damage, reduces spontaneously, is regular and continuous, gets tense gradually, and leads to a pleasant incident which is childbirth [2]. This pain is even referenced in the Bible verse where God cursed the woman to suffer labour pain when the early man disobeyed God (Genesis 3: 16). Thus, labour pain is perceived as a normal life journey by most women and midwives [3, 4]. Labour pain is caused by uterine muscle contractions and pressure exerted by presenting part of the fetus against the maternal pelvis, urinary bladder, and bowel during the passage of the fetus through the birth canal [5, 6]. Loneliness, ignorance, unkind or insensitive treatment during labour, along with unresolved past psychological or physical distress, increases the chance that the woman will suffer. Many women in labour each day in sub-Saharan Africa particularly in Nigeria, childbirth is experienced not as a joyful event but as a sad experience due to midwives' attitude towards the laboring woman who shouts and yells at laboring women especially if she screams cries or complains of labour pain [7]. Labour pain can be managed using either pharmacologic analgesia or non-pharmacologic methods like hydrotherapy, acupuncture, continuous labour support and intradermal water blocks, transcutaneous electrical nerve stimulation, mobilization, heat and cold application, aromatherapy, massage, hypnosis, and psychological support [8, 9]. However, these management options can be real if the midwives have a good attitude towards labour pain relief and can sense the severity of the pain.

**Figure 1:**
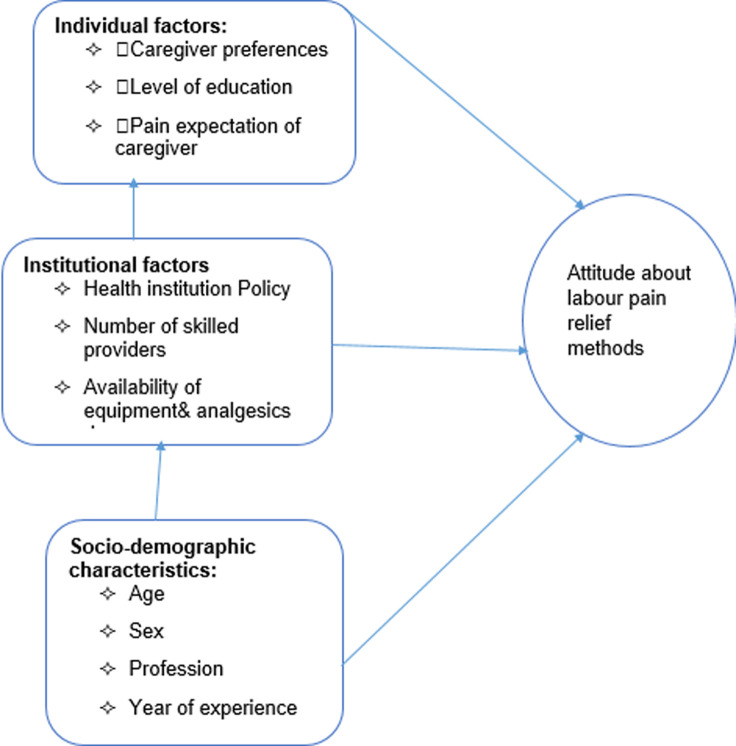
conceptual framework to assess the attitudes of labor pain relief methods and associated factors among obstetric caregivers at public health centers of East Gojjam zone, Amhara region, Ethiopia

Shreds of evidence showed that much health care professionals´ negative attitude towards effective management of labour pain, leaves many women and their babies to endure a reduced functional and psychological quality of life [10, 11]. According to the findings of studies conducted in Tigray region general hospitals and Amhara regional state referral hospitals showed the overall utilization of obstetric analgesia in labour pain management was found to be 43.3% and 40.1% respectively which only contribute to non - pharmacologic method [12-14]. According to the standard of midwifery care practice of 2013 in Ethiopia, the provision of physical and psychological support is a critical component to improve maternal satisfaction during childbirth and make it a joyful process [15]. As the studies conducted in Southeast Nigeria and Kenya reported that about 27% and 18% of laboring mothers have received pain relief methods during labour and delivery process respectively [16, 17]. Although delivery of the baby with a conscious and pain-free mother is one of the most exciting and rewarding moments of obstetric caregivers, most women in developing countries including Ethiopia still go through painful labour despite the availability of labour pain relief mechanisms [18-20]. As far as the researchers' knowledge is concerned, there are limited researches conducted in the study setting. Few local or international studies have been carried out on the current proposed study in Ethiopia. Thus, we were motivated to conduct this research on assessing the attitudes of obstetric caregivers and associated factors at public health centers of East Gojjam Zone, Amhara region, Ethiopia (Figure 1).

## Methods

**Study design:** an institutional-based cross-sectional study design was used.

**Study area and period:** the study was conducted in public health centers of the East Gojjam zone from March 1-30, 2018. East Gojjam is one of the administrative zones in the Amhara regional state of Ethiopia. It is bordered by the Oromia region in the south, on the west by the West Gojjam zone, on the north by the South Gondar zone, and on the East by the South Wollo zone. Debre Markos town is the capital city of East Gojjam zone which is 265Km far from Bahirdar, the capital city of Amhara region, and 299 Km from Addis Ababa, the capital city of Ethiopia. According to the projected census of 2007, conducted by the central statistical agency of Ethiopia (CSA), the zone has a total population of 2,153,937, an increase of 26.68% over the 1994 census, of whom 1,066,716 are men and 1,087,221 women; with an area of 14,004.47 square kilometers. East Gojjam zone has 22 woredas and 480 kebeles [21]. According to the East Gojjam zone health department annual first quarter yearly report of 2018, there were around 10 hospitals 100 health centers, and 423 health posts.

**Source population:** all health care providers(HCP) (midwife, nurse, and health officers) working in public health centers of the East Gojjam zone.

**Study population:** all HCP (midwife, nurse, and health officers) work in the delivery ward of the selected public health centers of the East Gojjam zone.

### Eligibility criteria

**Inclusion criteria:** all HCP (midwife, nurse, and health officers) worked in the delivery ward of randomly selected public health centers of the East Gojjam zone during data collection time.

**Exclusion criteria:** HCP (midwife, nurse, and health officers) comes to the labour ward for consultation during the study excluded from the study.

### Sample size determination

**The sample size for the outcome variable:** the sample size for the outcome variable was calculated based on a single population proportions formula as follows.


$$n={\left(Z\frac{a}{2}\right)}^{2}*\frac{p(1-p)}{d^{2}}$$

n = [1.96^2^*0.401(1-0.401)] / 0.05^2^= 369

Where: n is sample size

Zα/2 at 95%CI=1.96; d = is margin of error assumed to be 5%, = 0.05. P was the use of labour pain relief methods by obstetric caregivers = 40.1% according to study done in Amhara region referral hospital [13].

**Sample size for associated factors:** the sample size was calculated by double proportion formula using EPI info version 7 by associated factors from a study done in Amhara region referral hospitals [13]. Since the sample size calculated by outcome variable 369 is larger than the sample size calculated by associated factors, 254,369 was used to determine the final sample size (Table 1). Due to the source population (N=1433) is < 10,000 the sample was adjusted by reduction formula.

**Table 1:** sample size calculation to assess attitude of obstetric caregivers at health centers of East Gojjam Zone, Amhara region, Ethiopia, 2018 SG.C. (n =299)

Associated factors	Proportion	Power	CI	Calculated sample size
Lower educational level	P1=29.5% P2=47.3%	80%	95%	254
Inadequate knowledge	P1=52.5% P2=32.6%	80%	95%	212

P1=Proportion among unexposed, P2= Proportion among exposed.


$$n=\frac{n}{1+n/N}$$

n = 369/ (1+369/1433) =294

By considering 5% non-response rate, the minimum sample size for this study was 309.

**Figure 2:**
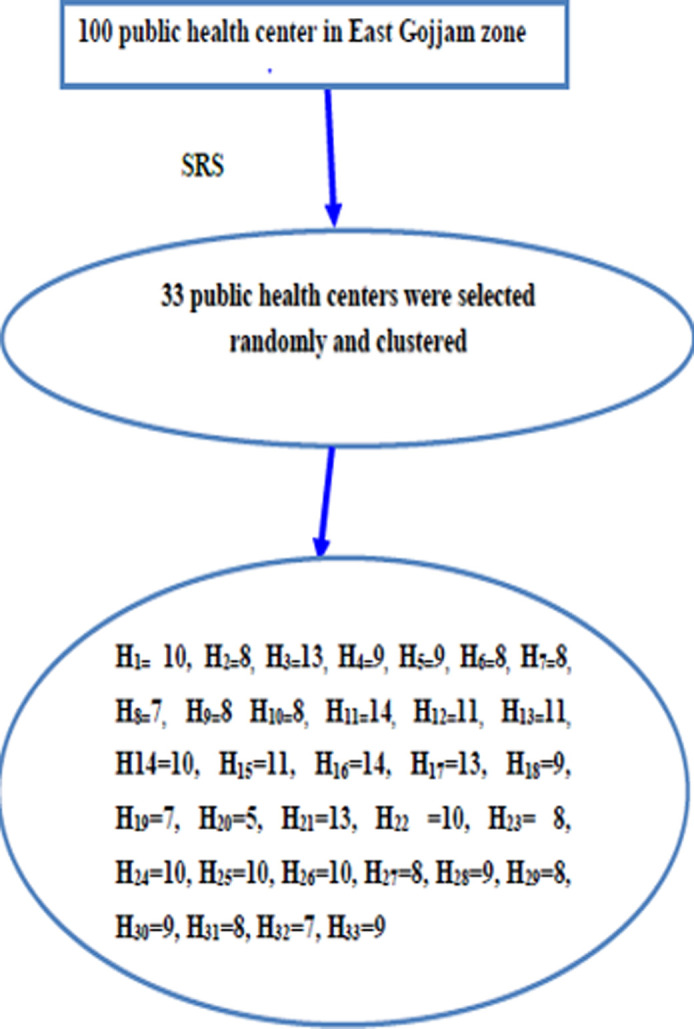
schematic presentation of sampling procedure to assess the attitudes of obstetric care givers at public health centers of East Gojjam Zone, 2018

**Sampling procedure:** a simple random sampling technique (using the lottery method) was used to select 33 public health centers (33% out of 100 public health centers) found in the east Gojjam zone. Then, all obstetric caregivers within the selected public health centers were included in the study by using the cluster sampling technique (Figure 2).

### Study variables

**Dependent variable:** the attitude of obstetric caregivers towards labour pain relief methods

### Independent variables

**Socio-demographic:** age, sex, profession, year of experience. **Individual factors:** caregiver preferences, level of education, pain expectation of caregivers. **Institutional factors:** health institution policy, number of skilled providers, availability of equipment and analgesics drugs.

### Operational definition

**Favorable attitude:** OCGs who answered greater than or equal to the mean value of attitude-related labour pain relief method questions. Those OCGs who answered = 8.18 from total attitude-related questions were considered as OCGs who had a favorable attitude about labour pain relief methods.

**Unfavorable attitude:** OCGs who answered less than the mean value of attitude-related labor pain relief method questions. Those OCGs who answered < 8.18 from total attitude-related questions were considered as OCGs who had an unfavorable attitude about labour pain relief methods.

### Method of data collection and tools

A semi-structured self-administered questionnaire with multiple choice was used to collect data from study participants. The questionnaire was adapted from reviewed kinds of literature [12, 13, 22] with modification and contextualized into local settings. The questionnaires consist of 3 parts, in which the first part was used to assess socio-demographic characteristics of obstetric caregivers, while the rest parts were used to assess the attitude towards labour relief methods and institutional factors affecting the use of labour pain management. The questionnaire was designed in English to be understood by all study participants. A self-administered questionnaire was delivered to each obstetric caregiver during data collection time and requested to fill, honestly. Nine diploma nurses were recruited for data collection and two BSc midwives were the supervisor of the data collection procedure.

### Data quality control

The training was provided for data collectors and supervisors on objective, the benefit of the study, individual's rights, and informed consent for the common understanding of the study in general and the questioner in particular. A pretest was done in West Gojjam zone public health centers on 5% obstetric caregivers to modify the questioner two weeks before the actual data collection time. Regular supervision during data collection was made; the questionnaire was reviewed and checked for completeness, accuracy, and consistency by the principal investigators and supervisors.

### Data analysis

First, the questionnaire was checked manually for completion and any misfiled questions. Before the data entry code was given. Then data were entered into the computer by Epi data version 4.2. Software and exported to statistical package for social sciences (SPSS) version 23.0 software for analysis. Descriptive statistics were computed to determine frequencies and summary statistics (mean, standard deviation, and percentage) to describe the study population concerning socio-demographic and other relevant variables. Data were presented using tables. Initially, bivariate logistic regression was carried out to see the association of each of the independent variables with the outcome variables. Then multivariate logistic regression was carried out for variables with a p-value < 0.2 in bivariate logistic regression to determine 21 significant relationships between the dependent and independent variables. P-value of < 0.05 and 95% confidence level was used as a difference of statistical significance.

## Results

### Socio demographic characteristics of respondents

Out of the 309 sampled obstetric caregivers, 299 responded to the questionnaires, making a response rate of 96.8%. The mean age of the respondents was 28.96 (± SD = 4.195) years. The majority 194 (64.9%) of professionals were in the age group of 20-29 years. About 162 (54.2%) of the respondents, were males and 258 obstetric health caregivers (86.6%) were Orthodox Christians. Out of the total respondents, 31.1 % were midwives in a profession. Nearly half 149 (49.8%) of study participants were diplomas and 49.2% of had BSc degrees. Among respondents (61.9 %) had work experience of fewer than 5 years (Table 2).

**Table 2:** socio-demographic characteristic of obstetric caregivers working at labour ward in public health centers of east Gojjam zone, Amhara region, Ethiopia, 2018 SG.C. (n =299)

Characteristics	Frequency (n)	Percent (%)
**Age (in years)**		
20-29	194	64.9
30-39	97	32.4
≥40	8	2.7
**Gender**		
Male	162	54.2
Female	137	45.8
**Religion**		
Orthodox	258	86.3
Muslim	30	10
Protestant	10	3.4
Other©	1	0.3
**Profession**		
Health officer	71	23.8
Midwife	100	33.4
Nurse	128	42.8
**Level of education**		
Diploma	149	49.8
BSc degree	147	49.2
Masters	3	1
**Clinical experience (in years)**		
≤5	185	61.9
6-9	76	25.4
≥10	38	12.7

Other©= Catholic

### Institutional factors

From the respondents who know pharmacologic method type 153 (51.2%), 140 (46.8%), 102 (34.1%), and 93 (31.1%) of them reported diclofenac, paracetamol, Pethidine, and hyoscine are available in their health center respectively. Eighty-seven (29.1%) of study participants reported that allowing a companion as a choice laboring woman is not allowed by their health center and 87 % of respondents reported that they didn't get any special training on management of labour pain.

### The attitude of obstetric caregivers towards labour pain relief methods

Regarding the attitude of obstetric caregivers, 171 (57.2%) of respondents had a positive attitude, whereas 128 (42.8%) of them had a negative attitude towards managing labour pain. About 258 (86.3%) of the total study participants believed managing labour could help the laboring woman to cope with labour pain, but only 171 (57.2%) of study participants perceived that every mother's pain during labour should be managed. Among total respondents,167 (55.9%) of them believed that the pharmacologic labour pain relief method (analgesic) is not necessary for managing labour pain. Of the respondents, 114 (38.1%) believed as labour pain is natural, and the mother has to face it, but nearly three fourth of 220(73.6%) of them consider managing labour pain as their responsibility of care for laboring mother. More than half of 173 (57.9%) of the study participants reported that they will administer the labour pain relief method for laboring women if they had resources at their hand.

### Factors associated with attitude of obstetric caregivers towards labour pain relief methods

The attitude of obstetric caregivers towards labour pain relief methods was cross-tabulated with socio-demographic factors, institutional related factors, and service-related factors, and the following ones were significantly associated with the outcome variable. In the bivariate logistic regression Profession, knowledge, training, and companion were factors associated with an attitude of obstetric caregivers about labour pain relief methods. But only knowledge, training, and Companion remained significantly associated in multivariable logistic regression (Table 3).

**Table 3:** bivariate and multivariate analysis of factors associated with attitude of obstetric caregivers towards labour pain relief methods east Gojjam zone, Amhara regional state, in Ethiopia, 2018. G.C.(n=299)

	Attitude of obstetric caregivers		COR (95%CI)	AOR (95%CI)	p value
Characteristics	Favorable	Unfavorable			
	Frequency (n)	Frequency (n)			
**Profession**					
Midwife	62(66.7%)	31(33.3%)	1.780 (1.068-2.966)	1.255(0.718-2.196)	0.425
Others	109(52.9%)	97 (47.1%)	1.00	1.00	
**Companion**					
Yes	136(64.2%)	76(35.8%)	2.659 (1.593-4.437)	1.834 (1.055-3.189)	.031*
No	35(40.2%)	52(59.8%)	1.00	1.00	
**knowledge**					
Adequate knowledge	118(72.8%)	44(27.2%)	4.250 (2.609-6.924)	3.785(2.251-6.365)	0.000*
Inadequate knowledge	53(38.7%)	84(61.3%)	1.00	1.00	
Training					
Yes	30(77%)	9(23%)	2.813(1.285-6.161)	2.923(1.266-6.749)	0.012*
No	141(54.2%)	119(45.8%)	1.00	1.00	

**Others:** Health officers, Nurse

## Discussion

For a country like Ethiopia with a health policy of improving the quality of maternal services, it is important to assess the use of labour pain relief methods among obstetric caregivers to manage labour pain, which contributes to the quality of intrapartum care for a laboring woman. This facility-based cross-sectional study was conducted to assess the attitude of obstetric caregivers towards labor pain relief methods and associated factors in the East Gojjam zone, Amhara region. This study gives important findings regarding current activities carried out to manage labor pain and possible improvement measures that could be implemented to enhance the quality of maternal health services to meet the need of laboring women. According to this study, 57.2% of obstetric caregivers had a positive attitude, which is consistent with a study done in Tigray region general hospitals (56.7%) [12]. But this result is higher than a study done in Amhara region referral hospitals (26.4 %) [13]. This may be due to the difference in study time and changing awareness among obstetric caregivers about the necessity of labor pain relief methods on labor pain management for a laboring mother through time.

In this study, the odds of having a favorable attitude towards labor pain management among OCGs who had adequate knowledge was 3.785 times higher than those who had inadequate knowledge. This finding is supported by the studies done in Amhara region referral hospitals [13] and Southern Ethiopia [14]. This may be due to the association of increase in knowledge, influence the attitude of individuals´ and greater knowledge inevitably leads to an enhanced attitude of individuals towards labor pain management. This study also revealed that the odds of having a favorable attitude about labor pain management among OCGs who provide labor support care in public health centers that allow companions were 1.834 times higher than those that didn't allow companions. This result is in agreement with the study done in Australia [23]. The possible explanation may be as a result of the positive influence of companion presence on obstetric caregivers´ behaviors in terms of sharing information related to the care given to the laboring woman. The result of this study reported that the odds of having a favorable attitude towards labor pain management among OCGs who had training about labor pain management were 2.923 times higher than those who had not been trained about labor pain management. This finding is in concur with the study conducted in Amhara regional state, Ethiopia [24]. This may be due to the effectiveness of the training to promote the desired changes and changing attitudes due to getting new information and knowledge about labor pain relief methods through the training.

**Limitation of the study:** since the study was a cross-sectional study it did not address the cause and effect relationship of the factors and the outcome variables.

## Conclusion

Even though most obstetric caregivers expect labor pain as severe pain in this study, most laboring women go through painful labor. The attitude of obstetric caregivers towards labor pain relief methods was affected by training, companion and knowledge of obstetric caregivers about labor pain relief methods. Recommendations: East Gojjam zone health department: prepare special training, with task-oriented refreshment course, special emphasis on strengthening obstetric caregivers' knowledge and attitude towards the use of labor pain relief methods through communicating with other concerned bodies. Non-governmental organization: participate in providing short-term training issues related to labour pain and labour pain relief methods for obstetric caregivers in East Gojjam Zone. Obstetric caregivers: empower women to ask labour pain relief services and update their knowledge and attitude about labour pain relief methods. Researcher: researcher should examine the use of labour pain relief methods from maternal request point of view.

### What is known about this topic

- Knowledge of obstetric caregivers towards labour pain relief methods;
- Factors influencing knowledge of obstetric caregivers towards labour pain relief methods.

### What this study adds

- Attitudes of obstetric caregivers towards labour pain relief methods;
- Factors affecting Attitudes of obstetric caregivers towards labour pain relief methods.
